# Supplementary material for: PSD95 as a New Potential Therapeutic Target of Osteoarthritis: A Study of the Identification of Hub Genes through Self-Contrast Model
Source: Int J Mol Sci. 2023 Sep 28;24(19):14682. doi: 10.3390/ijms241914682 (PMC10572132; doi:10.3390/ijms241914682)
Supplement: Supplementary file 1 [file ijms-24-14682-s001.zip › ijms-2597373-supplementary.pdf]

Table S1. Primer sequences for RT-qPCR.

Genes	Forward primer (5'-3')	Reverse primer (5'-3')
DLG4	TCGGTGACGACCCATCCAT	GCACGTCCACTTCATTTACAAAC
MMP13	ACTGAGAGGCTCCGAGAAATG	GAACCCCGCATCTTGGCTT
IL-1β	ATGATGGCTTATTACAGTGGCAA	GTCGGAGATTCGTAGCTGGA
IL-6	ACTCACCTCTTCAGAACGAATTG	CCATCTTTGGAAGGTTCAGGTTG
IL-8	TTTTGCCAAGGAGTGCTAAAGA	AACCCTCTGCACCCAGTTTTC

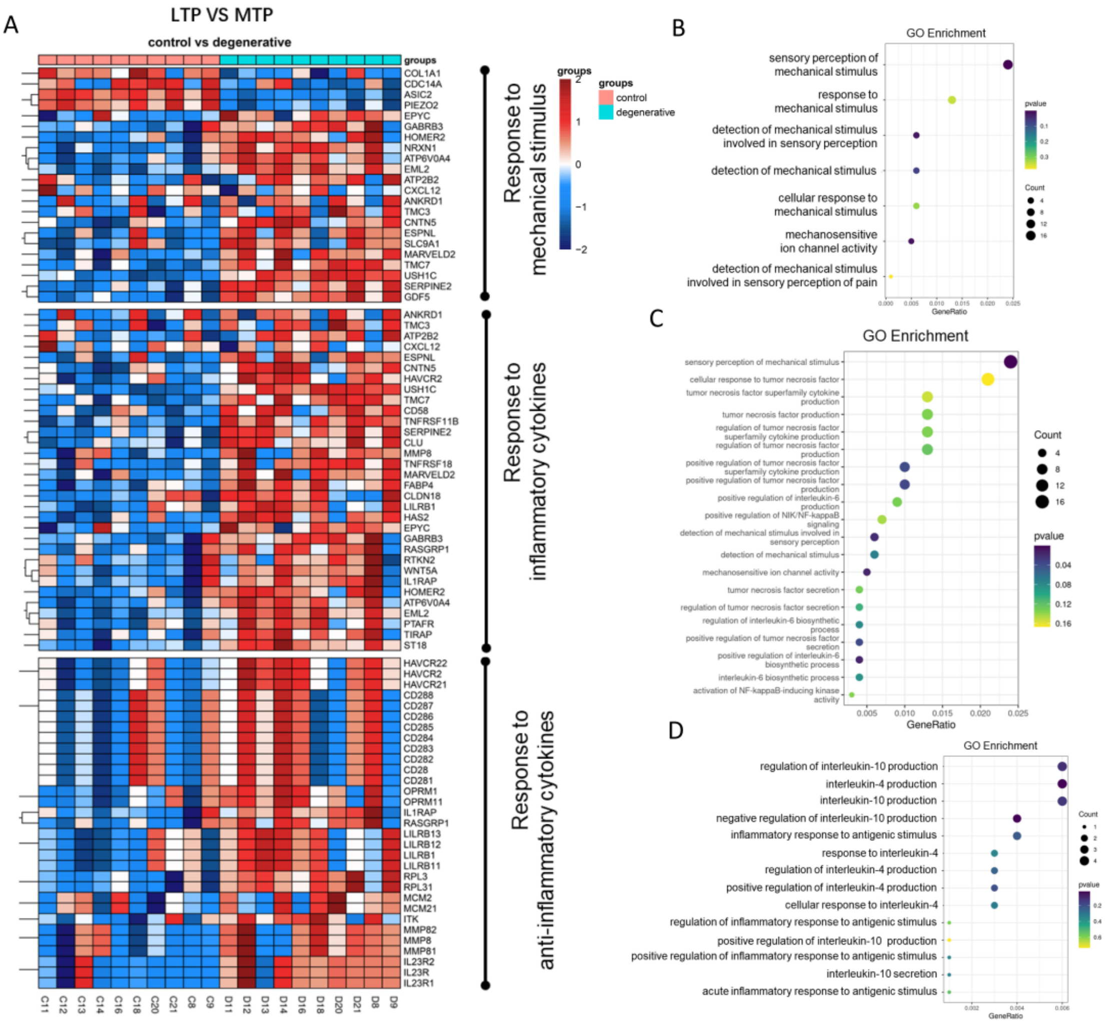

Figure S1. Transcriptome analysis of mechanical stress-related pathways and inflammatory response changes between LTP and MTP. (A) Heatmap of the expression profile of DEGs related to response to mechanical stimulus, response to inflammatory cytokines, and response to anti-inflammatory cytokines, which were upregulated in MTP compared to LTP. (B) The significantly overrepresented (adjusted p-value < 0.01) GO enrichment related to response to mechanical stimulus. (C) GO enrichment related to response to inflammatory cytokines. (D) GO enrichment related to response to anti-inflammatory cytokines.

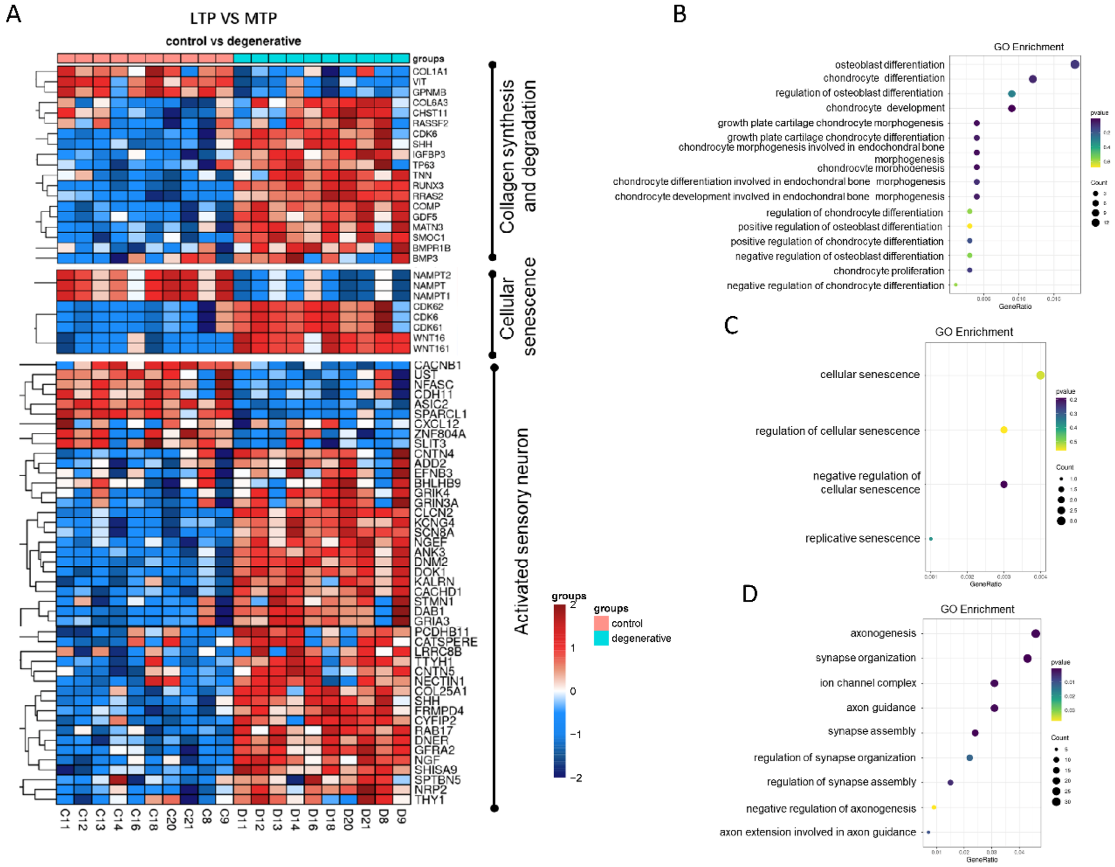

Figure S2. Gene-level characterization of collagen synthesis and degradation, cellular senescence, and activated sensory neurons between LTP and MTP. (A) Heatmap of the expression profile of DEGs related to collagen synthesis and degradation, cellular senescence, and activated sensory neurons, which was upregulated in MTP compared to LTP. (B) The numbers of significantly overrepresented (adjusted p-value < 0.01) GO enrichment related to collagen synthesis and degradation. (C) GO enrichment related to cellular senescence. (D) GO enrichment is related to activated sensory neurons.
